# The effect of mechanical strain on the Dirac surface states in the (0001) surface and the cohesive energy of the topological insulator Bi_2_Se_3_†

**DOI:** 10.1039/d1na00139f

**Published:** 2021-07-08

**Authors:** Soumendra Kumar Das, Prahallad Padhan

**Affiliations:** Department of Physics, Indian Institute of Technology Madras Chennai 600036 Tamil Nadu India Padhan@iitm.ac.in

## Abstract

The band gap (*E*_g_) engineering and Dirac point tuning of the (0001) surface of 8 QLs (quintuple layers) thick Bi_2_Se_3_ slab are explored using the first-principles density functional theory calculations by varying the strain. The strain on the Bi_2_Se_3_ slab primarily varies the bandwidth, modifies the p_*z*_ – orbital population of Bi and moves the Dirac point of the (0001) surface of Bi_2_Se_3_. The Dirac cone feature of the (0001) surface of Bi_2_Se_3_ is preserved for the entire range of the biaxial strain. However, around 5% tensile uniaxial strain and even lower value of volume conservation strain annihilate the Dirac cone, which causes the loss of topological (0001) surface states of Bi_2_Se_3_. The biaxial strain provides ease in achieving the Dirac cone at the Fermi energy (*E*_F_) than the uniaxial and volume conservation strains. Interestingly, the transition from direct *E*_g_ to indirect *E*_g_ state of the (0001) surface of Bi_2_Se_3_ is observed in the volume conservation strain-dependent *E*_g_. The strain on Bi_2_Se_3_, significantly modifies the conduction band of Se2 atoms near *E*_F_ compared to Bi and Se1, and plays a vital role in the conduction of the (0001) surface of Bi_2_Se_3_. The atomic cohesive energy of the Bi_2_Se_3_ slab is very close to that of (0001) oriented nanocrystals extracted from the Raman spectra. The strain-dependent cohesive energy indicates that at a higher value of strain, the uniaxial and volume conservation strain provides better stability than that of the biaxial strain (0001) oriented growth of the Bi_2_Se_3_ nanocrystals. Our study establishes the relationship between the strained lattice and electronic structures of Bi_2_Se_3_, and more generally demonstrates the tuning of the Dirac point with the mechanical strain.

## Abstract 2

Tuning of the p_*z*_ – orbital population of Bi on the (0001) surface of Bi_2_Se_3_, which forms the Dirac cone at the *Γ* point, can be achieved under uniaxial, biaxial, or volume conservation strain.

## Graphical abstract

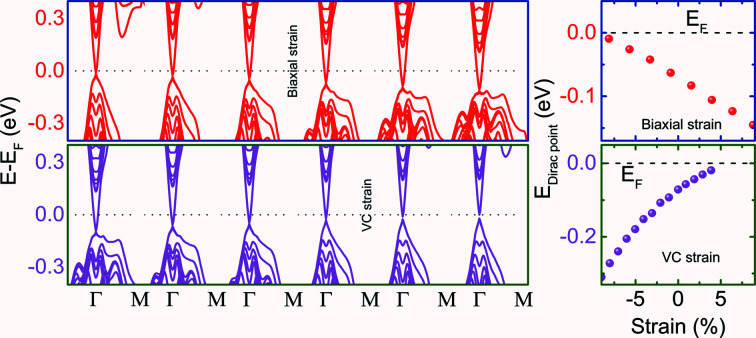

## 1. Introduction

The concept of topological insulators (TIs) has changed the conventional way of classifying solids based on symmetry breaking since it possesses symmetry protected topologically active surface states. TIs have created considerable attention among the condensed matter physics community, which behave as an insulator in its bulk but possess topologically active metallic surface states. TIs are characterized by a number known as the *Z*_2_ topological invariant, which can be calculated from the parity of the occupied bands for systems having inversion symmetry.^1^ Among the different compounds showing topological properties, Bi_2_Se_3_ is considered as the prototype of three-dimensional (3D) TIs and has been extensively studied over the last few years due to some of its unique features such as a simple band structure with a direct band gap (*E*_g_) of 0.3 eV, single Dirac cone at the *Γ* point^2^ and the helical spin texture of opposite spin helicity for the upper and lower Dirac cones.^3^ The tailoring of the topological surface states has been investigated using different external and internal agents to make the exotic topological phenomena viable for potential applications.^4,5^ Some of the widely studied agents are stress, electromagnetic field, chemical substitutions, stacking defects,^6^*etc.* In particular, strain plays a significant role in tuning the physical properties of TIs. For example, the first-principles density functional theory (DFT) calculations show the shift of the Dirac point of the helical surface states,^7^ enhancement or destruction of surface states,^8^ improvement in thermoelectric properties,^9^ decrease in the bulk carrier density,^10^ decrease in the coulombic gap, and increase in the strength of the spin–orbit interaction^11^ in Bi_2_Se_3_. Using van der Waals DFT and semi-classical Boltzmann theory, the thermoelectric figure of merit of a single quintuple layer (QL) of Bi_2_Se_3_ is found to be 0.27, which is more than the bulk value 0.10, and can be further increased to 0.30 by introducing 2.5% compressive strain.^12^ DFT calculations have also established that strain plays a crucial role to tune the ground state of the monolayer (ML) In_2_Se_3_. The strain fully suppresses the polarization in ML-In_2_Se_3_ and causes out-of-plane polarization in 2D non-polar materials, *i.e.*, ML-Bi_2_Se_3_, ML-Bi_2_Te_3_, ML-Sb_2_Te_3_ and ML-Sb_2_Se_3_.^13^ Strain engineering also predicts the occurrence of new 3D TI compounds with anti-perovskite structures in ternary cubic centrosymmetric compounds (M_3_N)Bi where M = Ca, Sr, Ba.^14^ Strain engineering plays a unique role in controlling the band structure and influences the Dirac point energy, Fermi velocity, metallic character, and the topology of the Bi based chalcogenide compounds. *Ab initio* calculations suggest the modification in the band structures, the absence of bulk-free carrier states, enhancement in the *E*_g_ opening in highly strained Bi_2_Se_3_ films that create the possibilities for a TI-based field-effect transistor.^15^

Inspired from the theoretical prediction of the possible tuning of bulk band gap and surface states of Bi_2_Se_3_ by the elastic strain, the strain has been applied on Bi_2_Se_3_ using different configurations, such as substrate-induced strain, stretching of the flexible substrate, doping, and the intercalation. Chae *et al.* have grown Bi_2_Se_3_ films on graphene/SiO_2_ and SiO_2_ surface.^16^ The 3 QLs thick film of Bi_2_Se_3_ grown on graphene/SiO_2_ exhibits strain values of 5.4%, while the film on SiO_2_ surface shows 7.6%. Interestingly, the strain on 2 QLs Bi_2_Se_3_ films is much lower than that of the 3 QLs film, implying that the interfacial strain from the graphene substrate is much more dominant as the thickness decreases within 3 QLs.^16^ However, the strain induced by the GaN substrate on Bi_2_Se_3_ is relatively low between 3% to 2.7%, although the lattice mismatch is 30%.^17^ The substrate-induced stress increases with thickness in the case of In_2_Se_3_ and decreases in the case of Bi_2_Se_3_ grown on sapphire because of the opposite lattice mismatch and different thermal expansion coefficients between the sample and the substrate.^18^ Flötotto *et al.* have grown ultrathin epitaxial Bi_2_Se_3_ films bonded onto conductive polyimide foils (Kapton).^19^ By using the strain holder, the strain has been applied to Kapton. This technique allows inducing around 2.1% in-plane strain on Bi_2_Se_3_. In addition, elemental doping in Bi_2_Se_3_ is a fundamental approach to introduce a mismatch in the effective ionic radii, *i.e.*, strain. Though there are several studies on elemental doping in Bi_2_Se_3_, the measurement of the induced strain is very scarce. Qi *et al.* have measured the strain induced by Mn in Bi_2_Se_3_ after doping. The doping of Mn introduces 6% strain in Bi_2_Se_3_.^20^ The other technique to introduce strain in Bi_2_Se_3_ is through intercalation of various species. The predominantly ionic nature of an intercalant requires either a change in the host lattice oxidation states or the presence of atomic vacancies to maintain charge neutrality, thus limiting the intercalant concentration.^21^ A zero-valent intercalant does not require a change in the oxidation state of the host lattice, thus allowing a high intercalation concentration.^22^ Using Rietveld refinement of the Bi_2_Se_3_ host lattice structure, Koski *et al.* have measured lattice parameters of Bi_2_Se_3_ and Cu-intercalated Bi_2_Se_3_.^21^ A 10% Cu intercalation in Bi_2_Se_3_ introduced strain of 9.66% in the plane and 0.35% along out-of-plane directions. Through an alternative measurement, electron diffraction confirmed, the introduction of 8.69% in-plane and 19.64% out-of-plane strain due to the 60% intercalation of Cu in Bi_2_Se_3_.^21^

Through low-temperature magneto-transport measurement, it is shown that the TI surface under the compressive strain of ±0.1% experiences a significant Dirac point shift (∓30 meV) as compared to the relaxed surface. The carrier mobility of TI is also increased for the surface under tensile strain.^23^ Although numerous reports available in the literature, a systematic study of strain engineering on surface states of Bi_2_Se_3_ is still lacking. In the present work, we have performed first-principles DFT calculations to study the electronic structure evolution of (0001) surface states of Bi_2_Se_3_ under the influence of in-plane and out-of-plane strain. The (0001) surface band structures show a substantial variation in the topological properties with the direction of strain (anisotropic behaviour).

## 2. Computational details

Band structure calculations were performed for (0001) Bi_2_Se_3_ surface using the Projector Augmented Wave (PAW) pseudopotential and plane-wave basis set as implemented in the Quantum Espresso (QE) package.^24^ Exchange-correlation potential was approximated through the Perdew–Burke–Ernzerhof general gradient approximation (PBE-GGA) functional. Further, the dispersion corrections were included through the semi-empirical Grimme-D2 van der Waals correction.^25^ The kinetic energy cut-off to fix the number of plane waves was taken as 50 Ry with the charge density cut-off 200 Ry. The Brillouin zone integration was carried out using a 6 × 6 × 1 Monkhorst pack *k*-point grid for structural relaxation, and a denser *k*-mesh 8 × 8 × 1 was used for electronic structure calculations. The convergence criterion for self-consistent energy was taken to be 10^−8^ Ry and the atomic positions were optimized until the force on each atom was lower than 10^−3^ eV Å^−1^. The surface calculations were performed using a slab model. An eight QLs slab of hexagonal Bi_2_Se_3_ with 40 atoms in the unit cell was generated using VESTA^26^ and XCrySDen software. A vacuum of 15 Å was added to the top QL to avoid the interaction among the surfaces of neighbouring slabs.

## 3. Experimental methods

Bi_2_Se_3_ nanostructures were prepared at 250 °C by the chemical hot injection method. Commercial grade Bi_2_O_3_ (99.999%) and elemental Se (99.99%) powders were chosen as precursors, while 1-octadecane and oleic acid were used as solvent and reducing agent, respectively. To prepare the Bi precursor, 1 mmol of Bi_2_O_3_ was mixed with 5 ml of oleic acid and 20 ml of octadecane in a round bottom flask, mixed vigorously using a magnetic stirrer for 60 min, then heated to 100 °C and were kept at that temperature for 30 min for degassing. Then, the Bi precursor was prepared at 250 °C for 360 min under a nitrogen flow. In a typical synthesis, the elemental Se powder was mixed in 15 ml of octadecane in another round bottom flask, heated to 250 °C, after degassing at 100 °C, then the Bi-precursor solution was injected into the flask. Finally, the solution was cooled to room temperature, washed with ethanol and hexane (1 : 3 ratio) several times, centrifuged, and finally, the product was dried at 60 °C.

The phase of these nanoplates was confirmed using a Rigaku Smart lab X-ray diffractometer with Cu-K_α_ radiation (*λ* = 1.5405 Å) (see Fig. S2†). The morphological studies were carried out by using a high-resolution transmission electron microscope (HRTEM) (FEI Tecnai-G^2^ T20) with an operating voltage of 200 kV (see Fig. S3†). The Raman spectra were recorded on a Jobin-Yvon LabRAM HR800UV spectrometer instrument equipped with a highly efficient thermo-electrically cooled charge-coupled device (CCD). The spectra were taken at different temperatures in the backscattering configuration using a 632 nm emission line of a He–Ne laser with laser power of 65 μW on the sample surface.

## 4. Result and discussion

**Fig. 1:**
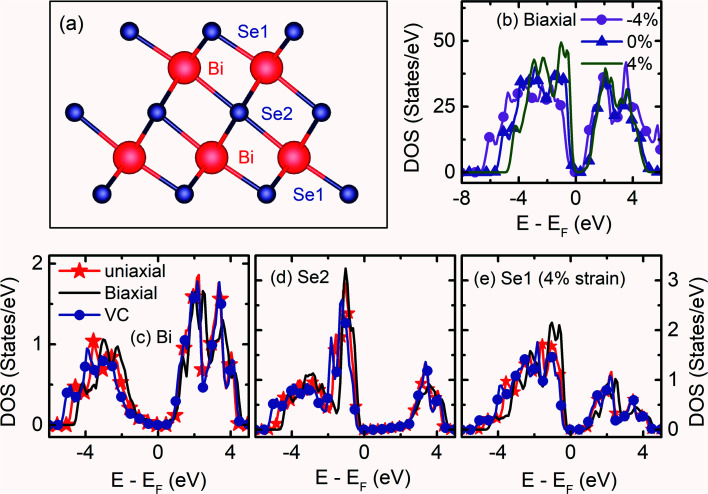
(a) Schematic of a single quintuple layer of Bi_2_Se_3_ indicating the order of the atomic arrangement. (b) The total density of states of the (0001) surface of Bi_2_Se_3_ without spin–orbit coupling under *ε*_*a*_ strain. Projected density of states of (c) Bi, (d) Se2 and (e) Se1 atoms on the (0001) surface of Bi_2_Se_3_ without spin–orbit coupling under tensile *ε*_*c*_, *ε*_*a*_ and *ε*_*c*_ = −*ε*_*a*_ strain.

Bulk Bi_2_Se_3_ belongs to the tetradymite type crystal with a hexagonal structure of the space group *R*3̄*m* (*D*^5^_3d_). Bi_2_Se_3_ possess a layered structure along the hexagonal *c*-axis [Fig. S1a†]. The conventional unit cell of the hexagonal Bi_2_Se_3_ contains 15 atoms, with five atomic layers arranged in a particular order called ‘Quintuple Layer’ (QL) along the *c*-direction. There are three quintuple layers in a conventional hexagonal unit cell. Each QL consists of five atomic layers in the order Se1–Bi–Se2–Bi–Se1 [Fig. 1a]. The top and bottom Se1 atoms have the same Wyckoff positions and are in a similar interaction environment. The Se2 layer acts as the inversion center with two equivalent Bi and Se1 atoms [Fig. S1b†]. Due to the presence of inversion symmetry, the topological invariant can be calculated through the parity of occupied bands at ‘*Γ*’ point.^2^ Within a QL, the atomic interaction forms stronger covalent bonds, whereas the inter-quintuple layer interaction is weak van der Waals type. The thickness of each QL is about 0.96 nm.^27^

The Slab model has been used for the surface states calculations of (112̄1) Bi_2_Se_3_.^28^ The calculations show that the energy gap at the Dirac point is closed for slab thickness larger than 6 QLs of Bi_2_Se_3_. However, the (0001) surface *E*_g_ of the Bi_2_Se_3_ is vanishingly small but finite for 6 QLs thick slab and becomes ideally zero with slab thickness above 6 QLs.^29^ Therefore, we have chosen an 8 QLs thick slab with 40 atoms to minimize the computational error and acquire reliable results. The unstrained 8 QLs slab constructed using the lattice parameters of the hexagonal-shaped nanocrystals of Bi_2_Se_3_ prepared by adopting a chemical hot-injection method is used.^29^ The crystal structures of these nanocrystals are established from the Rietveld refinement analysis, which confirmed the *R*3̄*m* (*D*^5^_3d_) crystallographic group of Bi_2_Se_3_. The refinement of the X-ray diffraction profile with a high degree of precision reveals the cell parameters; *a* = 4.136 Å and *c* = 28.59 Å of Bi_2_Se_3_ [Fig. S2†].^29^ The hexagonal shape morphology and the (0001) orientation of nanocrystals are further confirmed by the transmission electron microscopy measurement [Fig. S3†]. Thus, the reference lattice parameters of 8 QLs hexagonal Bi_2_Se_3_ slab with 15 Å vacuum layer are *a*_ref_ = 4.136 Å and *c*_ref_ = 100.9 Å. These lattice parameters are varied individually or simultaneously to apply strain on the slab and study the band structure of the strained Bi_2_Se_3_ (0001) surface. The out-of-plane lattice parameter ‘*c*’ is varied to apply uniaxial strain, which can be defined as 
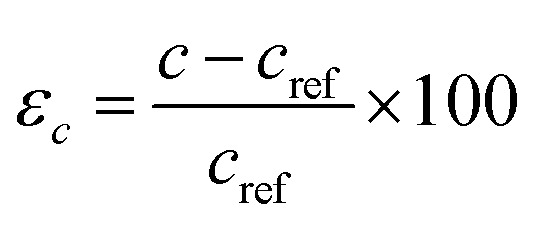
. The in-plane lattice parameter ‘*a*’ is changed for the application of biaxial strain 
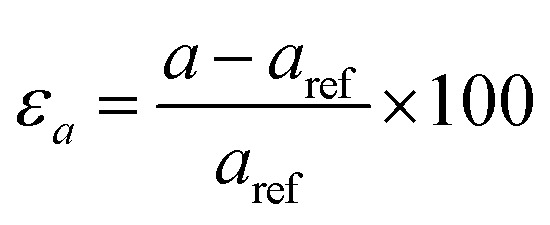
. While the *ε*_*a*_ = −*ε*_*c*_ strain with the conservation of volume 
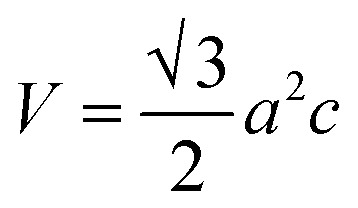
 is generated by varying both ‘*a*’ and ‘*c*’, simultaneously. The strain on the Bi_2_Se_3_ slab primarily varies the band width, which is reflected in the density of states (DOS).

Before the studies of the (0001) surface band structure of Bi_2_Se_3_, the bulk band structure calculations are carried out after relaxing the atomic coordinates to the minimum energy. The bulk band structure shows a direct *E*_g_ ≈ 0.19 eV [Fig. S4a†], which is very close to the reported experimental^30–32^ and theoretical^33,34^ values. The kinetic energy in the Hamiltonian without spin–orbit coupling term does not depend on the spin degrees of freedom. However, in real crystals, the spin and orbital motions of the electrons couple together. The incorporation of SOC in the band structure calculation of Bi_2_Se_3_ removes the spin degeneracy with the metallic edge or surface states are consistent with the high-momentum-resolution angle-resolved photoemission spectroscopy experiment.^35^ Interestingly, after including the SOC, the band structure shows an expansion of the *E*_g_ = 0.38 eV [Fig. S4b†], the bulk *E*_g_ consistent with previous reports.^2,11,34,36^ The representative total DOS of the (0001) surface of Bi_2_Se_3_ with *ε*_*a*_ strain are shown in Fig. 1b. The compressive *ε*_*a*_ strain expands the band width, while tensile *ε*_*a*_ strain compresses the band width. The band width of Bi and Se represents the orbital occupancy, and the modification in the band width indicates the change in the electron density, orbital overlap and *E*_g_. The band width of the surface QL atoms due to the tensile *ε*_*a*_ = −*ε*_*c*_ strain expands, while that of the tensile *ε*_*a*_ compresses, as compared to the tensile *ε*_*c*_ strain [Fig. 1c–e]. The Bi atoms in the surface QL remain insulating irrespective of the nature or direction of the strain applied to the slab. However, Se atoms are very sensitive to the nature or directions of the strain applied to the slab. The Se atoms in the surface QL present away from the *E*_F_ for tensile *ε*_*c*_ and *ε*_*a*_ = −*ε*_*c*_ strain as compared to that of the tensile *ε*_*a*_ strain [Fig. 1c–e]. The conduction band (CB) band width of Se2 shrinks as well as shifts away from the *E*_F_ as compared to that of Bi and Se1, irrespective of the nature or direction of the applied strain. Thus, Se2 atoms play an essential role in the Bi_2_Se_3_ (0001) surface conduction.^36^

**Fig. 2:**
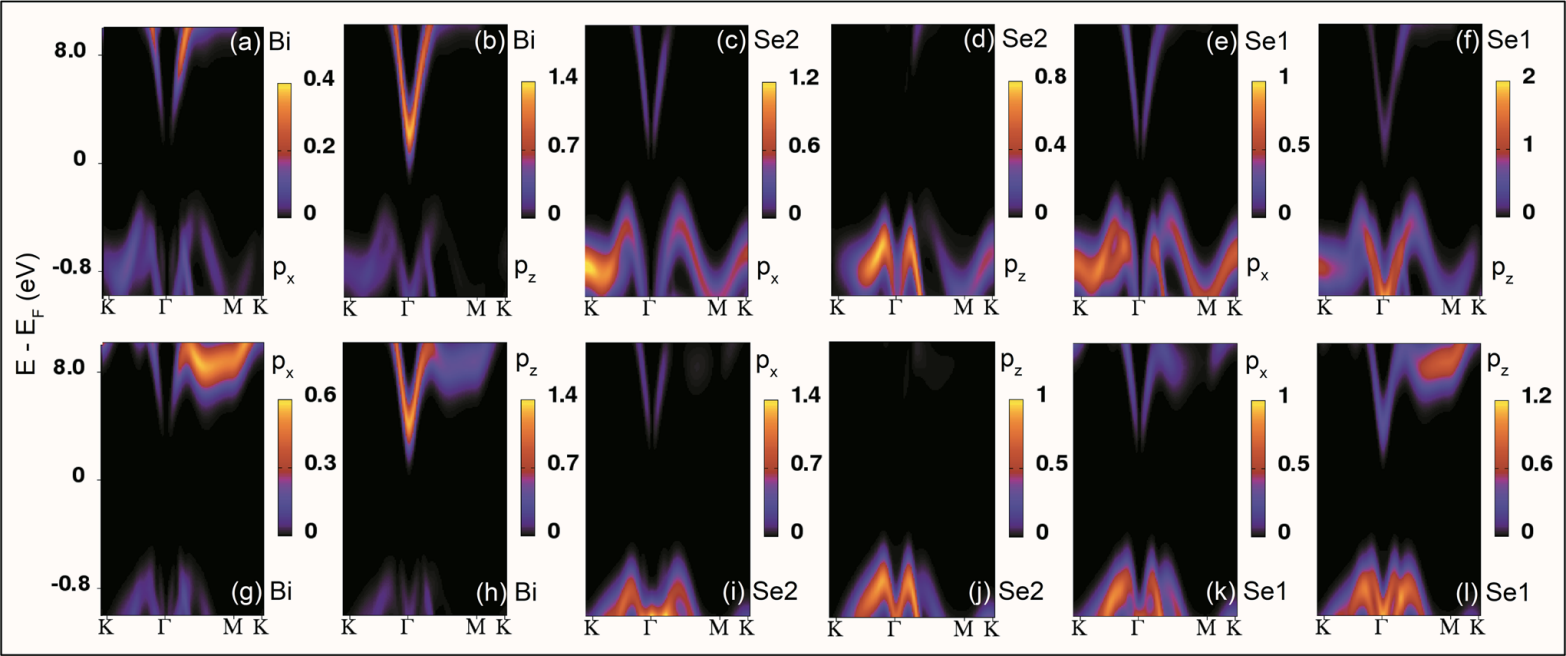
Projected band structures of the p_*x*_ and p_*z*_ orbitals of the Bi, Se2 and Se1 atoms on the (0001) surface of Bi_2_Se_3_ under uniaxial strain with −6% (a–f) and 6% (g–l).

The Bi atoms in Bi_2_Se_3_ contribute to the CB, while Se atoms contribute to the valence band (VB) near the *E*_F_. The p_*x*_ and p_*y*_ orbitals of both Bi and Se atoms are degenerate. The p_*x*_ and p_*y*_ orbitals of Bi atoms do not contribute at the *Γ* point and spread over the *Γ*–*M*–*K* path, while p_*z*_ orbital of Bi atoms disperse at and around the *Γ* point for the compressive *ε*_*c*_ strain on the Bi_2_Se_3_ slab [Fig. 2a and b]. As the compressive *ε*_*c*_ strain on the (0001) surface of Bi_2_Se_3_ decreases the p_*x*_ and p_*y*_ orbitals of Bi atoms move toward the *E*_F_ along the *Γ*–*M* path and the p_*z*_ orbital of Bi atoms move away from *E*_F_ [Fig. 2g and h]. Similar to Bi atoms, the p_*x*_ and p_*y*_ orbitals of Se atoms do not contribute at the *Γ* point, but populate over the *Γ*–*K* and *Γ*–*M*–*K* paths and p_*z*_ orbital populates around the *Γ* point due to compressive *ε*_*c*_ strain on the Bi_2_Se_3_ slab [Fig. 2c–f]. On decreasing the *ε*_*c*_ compression on the (0001) surface of Bi_2_Se_3_, the population near the *Γ* point due to the p_*x*_, p_*y*_ and p_*z*_ orbitals of Se atoms increase, and interestingly the p_*z*_ orbitals of Se1 atoms spread over the *Γ*–*M*–*K* path [Fig. 2i–l]. It is interesting to note that the orbital population of Bi and Se after the compressive *ε*_*a*_ strain on the Bi_2_Se_3_ slab is qualitatively similar to that of the tensile *ε*_*c*_ and the orbitals dispersion of Bi and Se due to the tensile *ε*_*a*_ strain is analogous to that of the compressive *ε*_*c*_ strain. The orbital population because of *ε*_*a*_ = −*ε*_*c*_ strain on the Bi_2_Se_3_ slab is similar to that of the *ε*_*c*_ strain.

**Fig. 3:**
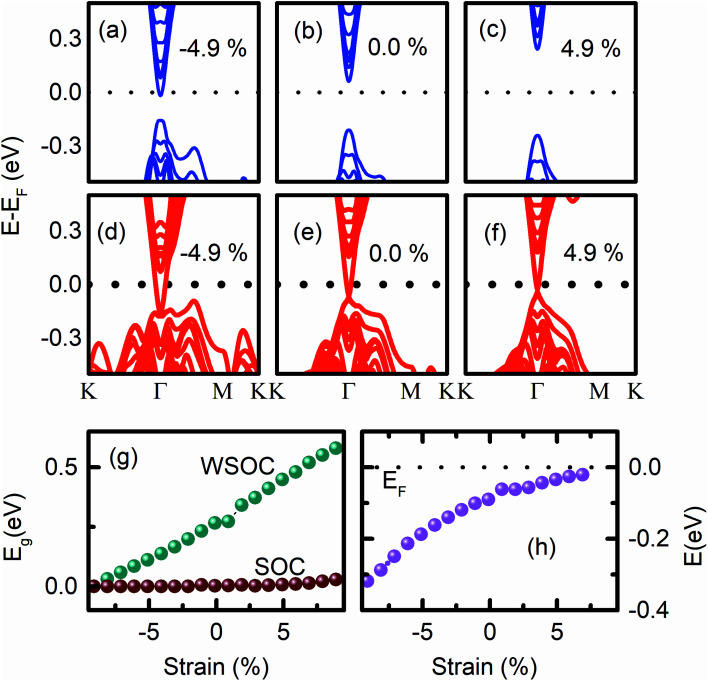
The (0001) surface band structures of Bi_2_Se_3_ without (a–c) and with SOC (d–f) under the *ε*_*c*_ strain. (g) The *ε*_*c*_ strain-dependent band gap of the (0001) surface of Bi_2_Se_3_ without and with SOC. (h) The position of the Dirac point energy with the variation of *ε*_*c*_.

The calculated *E*_g_ of the (0001) surface of unstrained Bi_2_Se_3_ slab without SOC (WSOC), *i.e.*, *a* = 4.136 Å, and *c* = 100.9 Å is ∼0.3 eV [Fig. 3b], which is closed to the reported value 0.26 eV for six QLs slabs.^12,37^ The corresponding band structures show an insulating direct *E*_g_ with the position of the conduction band minimum (CBM) near the Fermi energy (*E*_F_), but after including SOC, the surface shows metallic behaviour with the single Dirac cone at the *Γ* point [Fig. 3e], which is the hallmark for TIs.^2,38,39^ The occurrence of the Dirac point (DP) below *E*_F_ is consistent with previous reports of Bi_2_Se_3_.^40,35^

As the 8 QLs of the Bi_2_Se_3_ slab are compressed uniaxially by decreasing the ‘*c*’ value keeping ‘*a*’ and ‘*b*’ fixed, the direct *E*_g_ decreases gradually and becomes indirect at 4.9% compressive strain due to the formation of “M” shape at the VB maximum [Fig. 3a]. The *E*_g_ vanishes at a very large 10% compression [Fig. 3g]. This metallic state formation in the (0001) surface of the Bi_2_Se_3_ slab has been explained by the increase in the band width of p_*z*_ orbitals of Bi and Se in the valence band region.^36^ The indirect *E*_g_ in the (0001) surface of the Bi_2_Se_3_ has been observed previously for different combinations of lattice parameters under uniaxial strain.^41^ The compressive uniaxial strain has been imposed on the Sb_2_Se_3_ for the realization of its metallic state.^28^ On the other hand, as the tensile stress increases, *i.e.*, the value of ‘*c*’ increases above 100.9 Å, the *E*_g_ monotonically increases and moves the p_*z*_ orbital of Bi and Se atoms away from the *E*_F_, preserving the direct *E*_g_ [Fig. 3c and g]. Although achieving such a large value of strain is practically challenging, a recent experimental study demonstrates the occurrence of 9.6% strain along ‘*a*’ and 19.6% along ‘*c*’ through the intercalation of 60% zero-valent Cu into Bi_2_Se_3_ nanoribbons without disrupting the host lattice. The ‘*a*’ and ‘*c*’ lattice constants of Bi_2_Se_3_ increase from 4.14 ± 0.01 Å and 28.5 ± 0.01 Å to 4.54 ± 0.74 Å and 34.1 ± 0.76 Å, after the intercalation of Cu, respectively.^21^

The SOC makes the (0001) surface of Bi_2_Se_3_ conducting for an ample range of ‘*c*’ values. The Dirac cone appears deep inside the VB region at −0.16 eV due to the −4.9% *ε*_*c*_ strain, and p_*x*_ and p_*y*_ orbitals of Bi and the p_*z*_ orbital of Se1 atoms disperse along the *Γ*–*M*–K direction [Fig. 3d]. As the *ε*_*c*_ strain decreases, DP moves up towards *E*_F_ and appear very close to *E*_F_ (at −0.02 eV) for 4.9% *ε*_*c*_ strain [Fig. 3f], and the flat band feature in the VB is suppressed. The observed shift of DP energy in the band structure of the (0001) surface of Bi_2_Se_3_ by the variation of strain is consistent with previous studies on the strain-dependent DP energy of the (112̄1) surface of Bi_2_Se_3_.^8^ On further increasing the tensile *ε*_*c*_ strain, *i.e.*, increasing the ‘*c*’ value, the band structure opens a finite energy gap (0.05 eV) with the annihilation of the Dirac point [Fig. 3g]. The reports on the strain-induced annihilation of DP of the (0001) surface of the Bi_2_Se_3_ and the transition to a topologically trivial insulating phase is consistent with previous reports.^8,28^

The non-degenerate helical surface states of topological insulators provide many interesting topological phenomena and are the starting point for the Majorana excitations.^42^ The Dirac point should be at the *E*_F_ so that the conventionally confined states within the vertex are well separated from the Majorana-type excitations. The observed (0001) surface band structures of Bi_2_Se_3_ with the variation of *ε*_*c*_ strain confirmed that the uniaxial strain could be an effective tool to tune DP to be present at *E*_F_. The surface band structures show that the compressive strain moves DP away from *E*_F_, whereas the tensile strain moves the DP towards *E*_F_ [Fig. 3h]. In addition, the variation of *ε*_*c*_ strain provides the change of (0001) surface of Bi_2_Se_3_ from metallic-to-topological-to-normal insulator.^28,36^ The normal-to-topological insulator transition has been observed in β-As_2_Te_3_ by the application *ε*_*c*_ strain.^43^

**Fig. 4:**
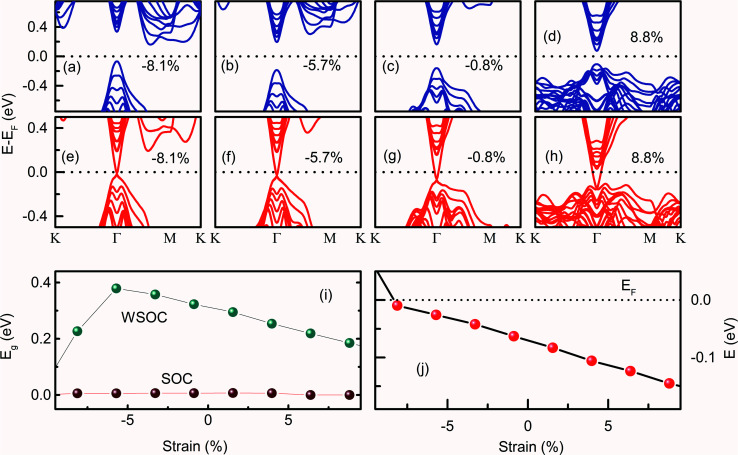
The (0001) surface band structures of the Bi_2_Se_3_ without (a–d) and with SOC (e–h) under the *ε*_*a*_ strain. (i) The *ε*_*a*_ strain-dependent band gap of the (0001) surface of the Bi_2_Se_3_ without and with SOC. (j) The position of the Dirac point energy with the variation of *ε*_*a*_.

The (0001) surface band structure of Bi_2_Se_3_ is investigated by applying the biaxial strain in the range of ±10%. The (0001) surface band structure of Bi_2_Se_3_ with −8.1% of *ε*_*a*_ strain exhibits the indirect *E*_g_ and the p_*x*_ and p_*y*_ orbitals of Bi atoms and p_*z*_ orbitals of Se atoms are dispersed near *E*_F_ [Fig. 4a]. As the compressive *ε*_*a*_ strain decreases, p_*x*_ and p_*y*_ orbitals of the Bi atoms disperse away from *E*_F_, while p_*z*_ orbitals of Bi atoms disperse towards *E*_F_, thus, the *E*_g_ becomes direct at −5.7% of *ε*_*a*_ strain [Fig. 4b]. In addition to the re-dispersion of orbitals, *E*_g_ increases from 0.28 eV to 0.38 eV, and the indirect-to-direct *E*_g_ transition occurs by the decrease of *ε*_*a*_ from −8.1% to −5.7%. On further decreasing the *ε*_*a*_ strain, *i.e.*, increasing the ‘*a*’ value, p_*z*_ orbitals of Bi and Se approaches towards *E*_F_, thus, the direct *E*_g_ decreases monotonically [Fig. 4c, d and i]. Interestingly, the biaxial strain is very sensitive to tune *E*_g_, the change of in-plane lattice parameter by 0.1 Å corresponds to approximately 2% variation in strain, which changes *E*_g_ by 0.02 eV. The change in *E*_g_ with *ε*_*a*_ is consistent with the variation of *E*_g_ in N-doped Sb_2_Te_3_, where an appropriate in-plane strain can enlarge the bulk *E*_g_.^44^ It is to be noted that the Bi_2_Se_3_ slab shows the direct *E*_g_ from −5.7% to 8.8% biaxial strain. The observation of direct *E*_g_ in a similar range of strain (−6% to +6% along the *x*-direction and −6% to 10% along the *y*-direction) is reported previously in Bi_2_Se_3_.^45^ On incorporating the SOC effect, the Dirac like dispersion is observed for the entire range of *ε*_*a*_ strain [Fig. 4e–h]. Interestingly, the DP is exactly at the *Γ* point at −8.1% of *ε*_*a*_ strain. As the *ε*_*a*_ strain decreases from −8.1%, the DP energy moves away from *E*_F_ [Fig. 4j]. It is interesting to note that both compressive and tensile *ε*_*a*_ strain could not open the *E*_g_ at the DP. The Dirac cone is preserved for the entire range of *ε*_*a*_ strain, which indicates that the topological properties remain preserved. A similar variation of tensile biaxial strain on the band structure of the (112̄1) Bi_2_Se_3_ slab was observed for one to six QLs thickness with the SOC effect.^36^ However, the result is limited to the tensile *ε*_*a*_ strain study and the effect of *ε*_*a*_ strain without SOC on the *E*_g_ of Bi_2_Se_3_ is very scarce.^36^

**Fig. 5:**
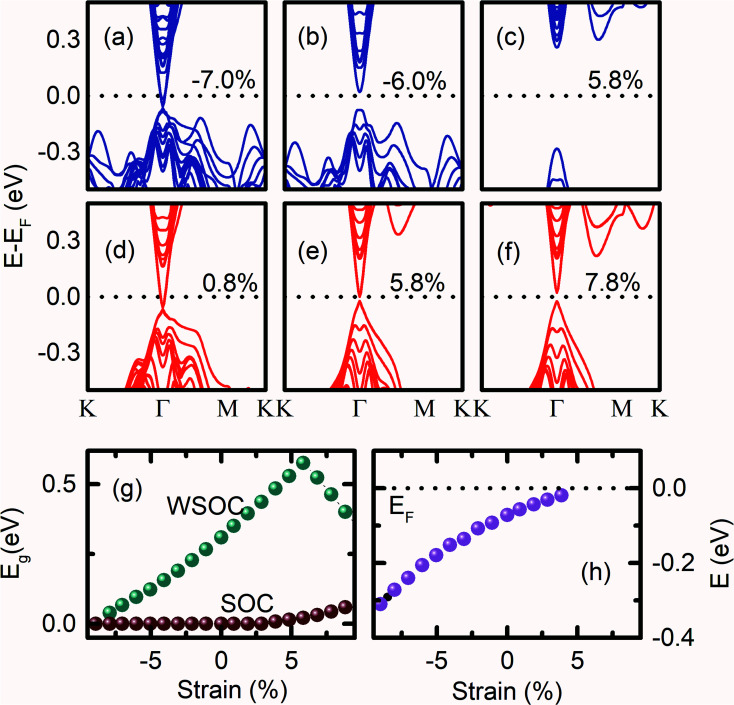
The (0001) surface band structures of Bi_2_Se_3_ without (a–c) and with SOC (d–f) under the volume conservation strain. (g) The volume conservation strain-dependent band gap of the (0001) surface of Bi_2_Se_3_ without and with SOC. (h) The position of the Dirac point energy with the variation of volume conservation strain.

The influence of strength of strain while keeping the volume of the Bi_2_Se_3_ slab constant, on the (0001) surface band structure of Bi_2_Se_3_ is also investigated. At −7.0% of compressive volume conservation strain, the (0001) surface of Bi_2_Se_3_ behaves like a degenerate semiconductor as p_*z*_ orbitals of Bi atoms cross the *E*_F_ [Fig. 5a]. However, the (0001) surface of Bi_2_Se_3_ behaves like a direct *E*_g_ semiconductor by reducing the compressive volume conservation strain to −6.0%, which moves p_*z*_ orbitals of Bi atoms away from *E*_F_ [Fig. 5b]. On further reducing the volume conservation strain from the compressive state to the tensile state, the (0001) surface gap increases up to 5.8% [Fig. 5c]. In addition, p_*x*_ and p_*y*_ orbital dispersions of Bi atoms spread towards *E*_F_ along the *Γ*–*M* direction. The (0001) surface of Bi_2_Se_3_ shows that the Dirac surface state feature with the Dirac point below the Fermi level for a smaller value of tensile strain [Fig. 5d]. Above 5.8% volume conservation strain, the (0001) surface gap decreases and becomes indirect as the p_*z*_ orbital of the Se atoms come closer to the p_*x*_ and p_*y*_ orbitals of the Bi atoms [Fig. 5g]. The decrease in the (0001) surface *E*_g_ at higher tensile strain could be due to the compressive *ε*_*a*_, which increases the band width. Note that the study of the band structure of Bi_2_Se_3_ with the variation of *ε*_*c*_ = −*ε*_*a*_ strain is very rare to compare our results. In the presence of SOC, the (0001) surface of Bi_2_Se_3_ is conducting if the strength of the applied strain with the conservation of the volume of the slab is between −7.9% to 4.9% [Fig. 5g]. The (0001) surface of Bi_2_Se_3_ is a normal semiconductor if the strain on the slab is larger than 4.9%. For *ε*_*c*_ = −*ε*_*a*_ > 4.9%, the band structures of the (0001) surface show an opening of the energy gap, which increases slowly with the increase in the strain [Fig. 5e and f]. For *ε*_*c*_ = −*ε*_*a*_ = −9.9%, DP is deep inside VB at −0.345 eV below *E*_F_ [Fig. 5h]. As the volume conservation strain decreases from −9.9%, DP moves towards *E*_F_ and appear at *E*_F_ for *ε*_*c*_ = −*ε*_*a*_ = 5.9%. On further increasing volume conservation strain, the Dirac cone feature of Bi_2_Se_3_ gets annihilated, and a finite *E*_g_ emerges at the *Γ* point. The position of the DP energy with the variation of *ε*_*c*_ = −*ε*_*a*_ strain is plotted in Fig. 5h, which is qualitatively very similar to the case of *ε*_*c*_ strain variation.

There are several DFT studies on the DP energy of Bi_2_Se_3_ surface,^7,8,28,40^ which interestingly indicates that the position of the DP energy depends on the orientation of the Bi_2_Se_3_ surface. For example, the DP exactly appears at *E*_F_ in the unstrained condition of (112̄1) oriented rhombohedral Bi_2_Se_3_.^2^ In contrast, the DP energy is located below and above *E*_F_ in (0001) and (11̄00) surfaces of the unstrained hexagonal Bi_2_Se_3_, respectively.^40,46^ The electrons are naturally accumulated on the surface of the real Bi_2_Se_3_ single crystals because of Se deficiency, *E*_F_ appears in the conduction band, which makes the detection of topological surface states difficult. Thus, the tuning of *E*_F_ position in the band gap is necessary for the realization of the topological surface states of Bi_2_Se_3_.^47^ Interestingly, our band structure calculations of the (0001) surface of Bi_2_Se_3_ show the tuning of the energy position of DP by the application of strain on Bi_2_Se_3_ slab. The DP energy can be tuned from −0.318 eV to −0.02 eV by changing uniaxial/volume conservation strain from −9.1% to 6.9%. As the uniaxial/volume conservation strain switches from compressive to the tensile state, the DP energy in CB moves towards *E*_F_. However, DP energy shifts from −0.009 eV to −0.145 eV by changing biaxial strain on the Bi_2_Se_3_ slab from −8.12% to 8.8%, the opposite effect of uniaxial/volume conservation strain. Moreover, the (0001) surface of Bi_2_Se_3_ becomes insulating, even in the presence of SOC for large tensile volume conservation strain, which indicates the reduction in the SOC strength. However, it is reported that the volume conservation strain on Bi_2_Se_3_ with the (11̄00) oriented surface lifts the spin degeneracy and shifts the DP energy away from the *Γ* point.^46^ For (112̄0) surface Bi_2_Se_3_, the DP energy appears above and below *E*_F_ for the tensile and compressive strain on the Bi_2_Se_3_ slab, respectively.^46^

Fig. 3–5 show that the occurrence of the redistribution of the orbital population of the (0001) surface due to the variable strain on Bi_2_Se_3_. The compressive *ε*_*a*_ strain increases the p_*x*_ and p_*y*_ orbitals overlap, which moves the orbital population towards the *E*_F_ along the *Γ*–*M*–*K* in the CB. Thus, the band gap becomes indirect and shows a peak in the band gap *vs.* strain graph [Fig. 4i]. A similar situation arises while applying tensile volume conservation strain, and a peak appears in the band gap *vs.* strain graph [Fig. 5g]. However, under compressive *ε*_*c*_ strain, the dispersion of p_*z*_ orbitals is dominated at the *Γ* point, and a direct band gap is observed in the +4.9% to +10% *ε*_*c*_ strain region. The band gap linearly varies with the *ε*_*c*_ strain without the application of SOC.

The result indicates that the (0001) surface of hexagonal Bi_2_Se_3_ is very sensitive to uniaxial strain, which can drive the (0001) surface state from metallic-to-topological-to-semiconducting nature with a maximum surface gap of 0.6 eV. The topological phase is observed in the range of ±6% variation of ‘*c*’. In contrast, the biaxial strain controls the dispersion of p_*x*_ and p_*y*_ bands, which toggles the (0001) surface of Bi_2_Se_3_ between the metallic and topological states. The topological state is observed for the variation of ‘*a*’ from −8% to 8%. Thus, the probability of achieving the topological surface states in the (0001) oriented hexagonal Bi_2_Se_3_ is higher for biaxial strain than the case of the uniaxial and volume conservation strain. Interestingly, biaxial strain could not create a finite energy gap at the Dirac point; hence, it does not play a significant role in destroying topological surface states. Therefore, the annihilation of the Dirac point largely depends on the out-of-plane interaction among the atomic orbitals of Bi and Se. The variation of p_*x*_, p_*y*_ and p_*z*_ orbitals with *ε*_*c*_ = −*ε*_*a*_ alleviate the (0001) surface of Bi_2_Se_3_ similar to the case of uniaxial strain. However, the tensile volume conservation strain distinguishes a transition from a direct *E*_g_ state to an indirect *E*_g_ state from the strain-dependent *E*_g_ plot without SOC. Overall, the Dirac cone can be tuned to *E*_F_ by the application of tensile uniaxial, compressive biaxial, and tensile volume conservation strain.

**Fig. 6:**
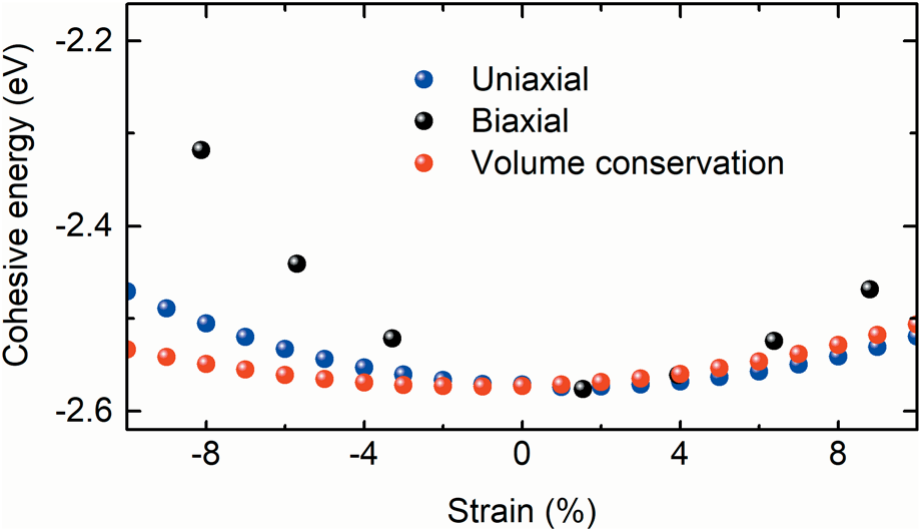
Atomic cohesive energy of the 8 QLs Bi_2_Se_3_ slab under the uniaxial, biaxial, and volume conservation strain.

Further, the atomic cohesive energy of the (0001) Bi_2_Se_3_ slab is calculated for different values of uniaxial, biaxial, and volume conservation strain. Fig. 6 shows the variation of cohesive energy per atom in the Bi_2_Se_3_ slab with the strain. The cohesive energy (*E*_coh_) is calculated using the expression;
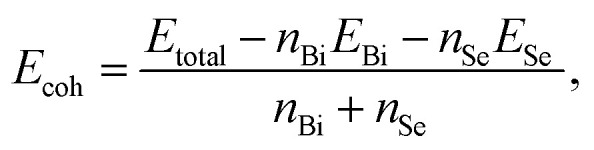
where *n*_Bi_ and *n*_Se_ are the number of atoms in the slab and *E*_Bi_ and *E*_Se_ are single isolated atom energies of Bi and Se, respectively. The *E*_coh_ of the reference structure is 2.57 eV, which is close to the theoretically calculated value and 2.97 eV.^48^

**Fig. 7:**
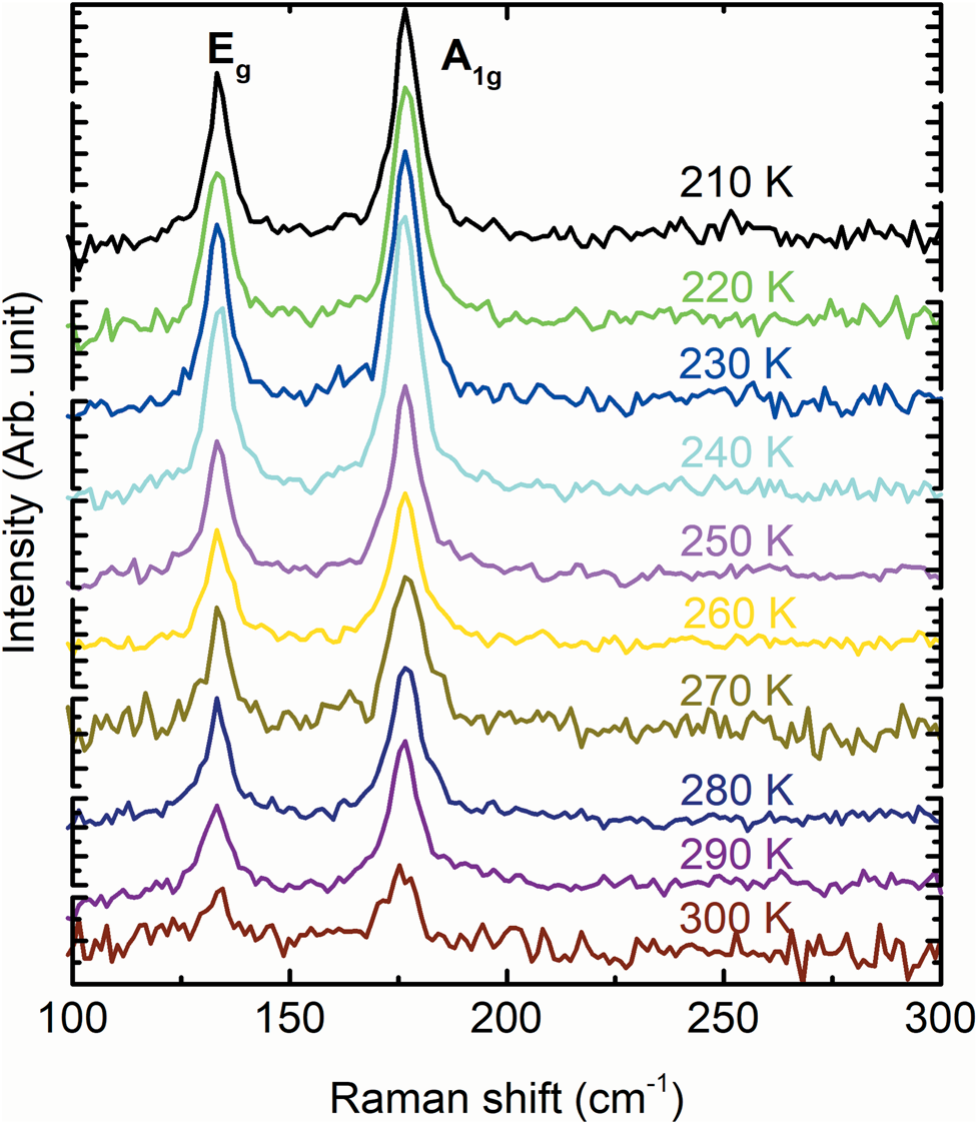
Raman spectra of the Bi_2_Se_3_ hexagon plates synthesized at 250 °C, recorded at various temperatures.

**Fig. 8:**
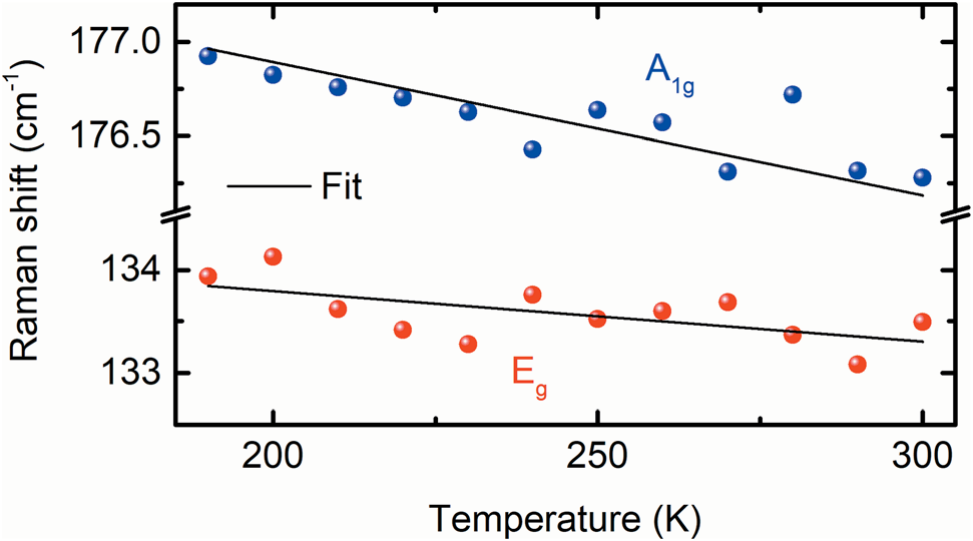
Temperature-dependent Raman shift of the *E*_g_ and A_1g_ modes of the Bi_2_Se_3_ hexagon plates synthesized at 250 °C. The solid line is the fit to the data.

To compare the theoretically calculated cohesive energy with that of Bi_2_Se_3_ nanocrystals, Raman spectra of Bi_2_Se_3_ nanocrystals were recorded at various temperatures above the Debye temperature (∼185 K)^49^ of the Bi_2_Se_3_ [Fig. 7]. The Raman spectra of these nanocrystals in the assessable Raman shift range of the Raman spectrometer exhibits a Raman line appearing at ∼131.2 and ∼173.4 cm^−1^, which correspond to the *E*_g_ and A_1g_ modes, respectively. The *E*_g_ mode appears because of the in-plane vibrations of Se and Bi atoms and A_1g_ mode appears from the out-of-plane vibrations of Se and Bi atoms.^50^ The peak position of different modes in Raman spectra of Bi_2_Se_3_ are extracted using the Lorentz fit and plotted in Fig. 8. The Raman shift (*ω*(*z*_b_,*T*)) of both modes varies linearly with temperature consistent with the following expression;^49^
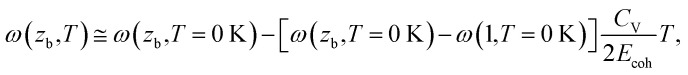
where *z*_b_ is the bulk coordination number, *C*_V_ is the three-dimensional specific heat per bond, and *ω*(1) is the vibrational frequency of an isolated dimer, which is the reference point for the optical redshift upon nanosolid and bulk. The above expression of *ω*(*z*_b_,*T*) is valid only for the material with negligible thermal expansion at high *T*, especially when the temperature is larger than the Debye temperature. The linear fit to the *ω*(*z*_b_,*T*) of the *E*_g_ and A_1g_ modes provides the *ω*(1) and *E*_coh_ of Bi_2_Se_3_ nanocrystals. The *ω*(1) is found to be 36.9 cm^−1^, which is very close to the value reported for Bi_2_Se_3_.^49^ The *E*_coh_ of Bi_2_Se_3_ nanocrystals calculated from the *ω*(*z*_b_,*T*) expression is 2.58 eV, which is larger than the values reported for Bi_2_Se_3_ nanostructures as 1.24 eV (ref. 49) and 1.37 eV.^51^ However, *E*_coh_ extracted from Raman scattering is very close to the value calculated from the first principles density functional theory. The cohesive energy remains close to 2.57 eV for ± 4% the variation of strain, which could be the foundation of the (0001) oriented growth of Bi_2_Se_3_ nanocrystals. Note that, the variation of cohesive energy is higher in biaxial strain as compared to the uniaxial and volume conservation strain state for the strain larger than ± 4%. The strain-dependent cohesive energy indicates that at a higher value of strain, uniaxial and volume conservation strain provides better stability than that of the biaxial strain (0001) oriented growth of Bi_2_Se_3_ nanocrystals. The strain (±4%) independent cohesive energy and the tuning of the Dirac point position with strain will shed light on achieving the dissipationless (0001) surface transport in Bi_2_Se_3_, which is the essential requirement for the application of topological insulators in topological quantum computation and low-power spintronic devices.

## 5. Conclusion

In conclusion, the (0001) oriented Bi_2_Se_3_ nanocrystals were prepared by a hot injection method using a nontoxic solvent. The atomic cohesive energy of these nanocrystals extracted from the temperature-dependent Raman spectra is 2.58 eV. By utilizing the lattice parameters of these nanocrystals, an 8 QLs thick Bi_2_Se_3_ slab is constructed to explore the effects of strain on the (0001) surface states and the cohesive energy calculated using the first-principles DFT calculations without and with the presence of SOC effects. The CB band width of the Se2 shrinks as well as shifts away from *E*_F_ compared to that of Bi and Se1 irrespective of the nature or directions of the applied strain. The band structures of the (0001) surface of Bi_2_Se_3_ show that Se2 is very sensitive to mechanical strain, and strain can tune the Dirac point energy. The Dirac cone feature of the (0001) surface of Bi_2_Se_3_ is preserved for the entire range of the biaxial strain. However, around 5% tensile uniaxial strain and even lower value of volume conservation strains annihilate the Dirac cone, which causes the loss of topological (0001) surface states of Bi_2_Se_3_. The biaxial strain provide ease in achieving the Dirac cone at *E*_F_ than uniaxial and volume conservation strains. The volume conservation strain distinguishes a transition from a direct *E*_g_ state to an indirect *E*_g_ state in the strain-dependent *E*_g_ plot without the SOC. The strain-dependent cohesive energy indicates that at a higher value of strain, uniaxial and volume conservation strain provides better stability compared to that of the biaxial strain (0001) oriented growth of Bi_2_Se_3_ nanocrystals. The application of mechanical strain on Bi_2_Se_3_ slab has a significant influence on the band width of ‘p’ orbitals of Bi and Se, *E*_g_ and Dirac point energy, which may provide a new pathway to control many physical properties of Bi_2_Se_3_ with strain and shade light on designing artificial topological materials for technological applications.

## Conflicts of interest

The authors declare no competing financial interest.

## Supplementary Material

NA-003-D1NA00139F-s001
