# Supplementary material for: The effect of mechanical strain on the Dirac surface states in the (0001) surface and the cohesive energy of the topological insulator Bi2Se3
Source: Nanoscale Adv. 2021 Jul 8;3(16):4816–25. doi: 10.1039/d1na00139f (PMC9416801; doi:10.1039/d1na00139f)
Supplement: NA-003-D1NA00139F-s001 [file NA-003-D1NA00139F-s001.pdf]

Electronic Supplementary Material (ESI) for Nanoscale Advances.
This journal is © The Royal Society of Chemistry 2021

Supporting Information

The effect of mechanical strain on the Dirac surface states in the (0001) surface and the cohesive energy of the topological insulator Bi_2Se_3

Soumendra Kumar Das [a] and Prahallad Padhan [a]

[a] Department of Physics, Indian Institute of Technology Madras, Chennai-600036, India

AUTHOR INFORMATION :

Corresponding Author :

padhan@iitm.ac.in

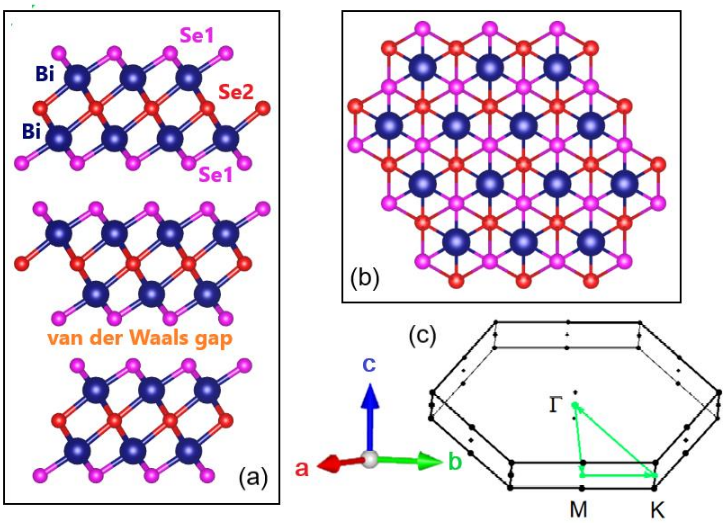

Fig. S1: (a) A unit cell of the $(10\bar{1}0)$ surface of the Bi_2Se_3 consisting of three quintuple layers (QLs). Each QL consists of five atomic layers in the order $Se1 - Bi - Se2 - Bi - Se1$. The QLs are bound by the weak van der Waals force. (b) The (0001) surface of the Bi_2Se_3 with $Se1 - Bi - Se2$ layers. (c) The 3-dimensional first Brillion zone of Bi_2Se_3 with space group $R\bar{3}m$ showing three nonequivalent time-reversal invariant momentum points Γ(0, 0, 0), M(0.5, 0, 0) and K(0.33, 0.33, 0).

Figure S1(a) shows the schematic figure for the layered structure of hexagonal Bi_2Se_3. The stacking order is along the 'c' direction in the sequence $Se1 - Bi - Se2 - Bi - Se1$, which constitutes a quintuple layer (QL). The covalent bond influences the atomic layers within a quintuple layer, but the interaction between two QLs are dominated by weak van der Waals forces. There are three crystallographic axes in the unit cell. It has a trigonal axis with threefold rotation symmetry (defined as the 'c' axis), a binary axis with twofold rotation symmetry (defined as the 'a' axis), and a bisectrix axis in the reflection plane (defined as the b axis)[1]. Due to the presence of inversion symmetry, the topological invariant can be calculated through the parity of occupied bands at 'Γ' point[2]. The top view of the (0001) surface of Bi_2Se_3 is sketched in Fig. S1(b), which shows the position of $Se1$, $Se2$, and Bi atoms.

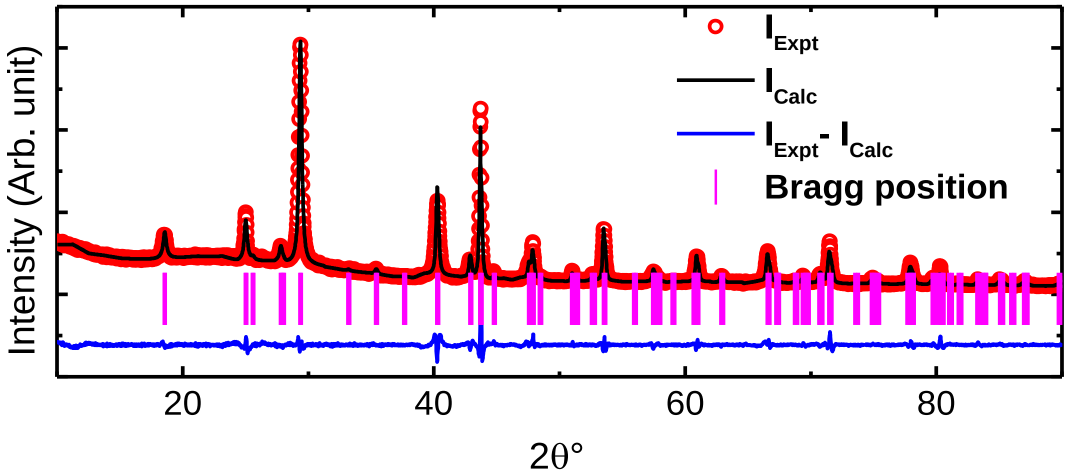

Fig. S2 $\theta - 2\theta$ x-ray diffraction pattern and Rietveld refinement profile of Bi_2Se_3 hexagon plates synthesized at 250 ℃.

Figure S2 shows the $\theta - 2\theta$ x-ray diffraction pattern of the Bi_2Se_3 nanocrystals, which resembles the characteristic peaks for the hexagonal crystal structure with space group $R\bar{3}m$. The x-ray diffraction patterns do not show any signs of the formation of parasitic or impurity phases. The phase identification is further confirmed from the Rietveld refinement analysis. The lattice parameters are estimated as $a = 4.136$ Å and $c = 28.59$ Å after the Rietveld refinement of the x-ray diffraction pattern to a high degree of precision[3].

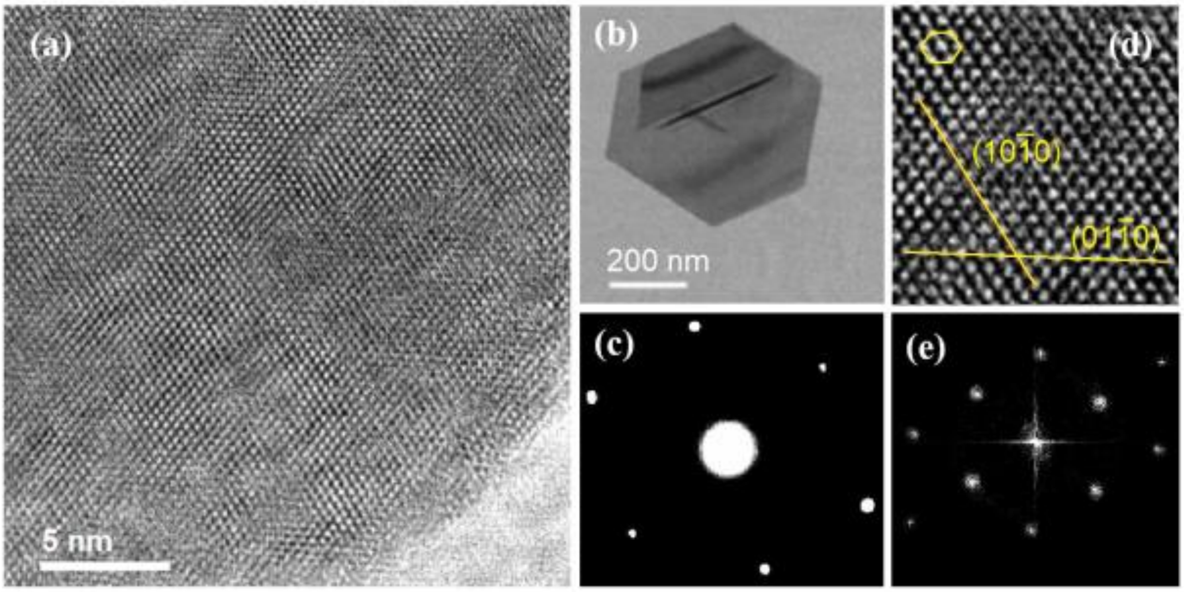

Fig. S3 (a) High-resolution transmission electron microscopy image, (b) a single hexagon plate with a flat surface and sharp edges, (c) selected area electron diffraction, (d) a section of zoom HRTEM image, and (e) Fast Fourier Transform of the HRTEM image of the Bi_2Se_3 nanocrystals synthesized at 250 ℃.

The high-resolution transmission electron microscopy ($HRTEM$) images of these hexagon plates of the Bi_2Se_3 (Fig. S3(a)) show lattice fringes with the hexagonal crystal structure, consistent with Figure S2. A typical hexagon plate of Bi_2Se_3 synthesized at 250 ℃ for 1 hr has a flat surface and sharp edges (Fig. S3(b)). This Bi_2Se_3 hexagon plate has a thickness of around 40 nm with a lateral dimension of 600 nm, which is consistent with the SEM image. The selected area electron diffraction ($SAED$) patterns show a two-fold symmetry reciprocal space diffraction spot pattern (Fig. S4(c)) and indicate the single-crystalline nature of the Bi_2Se_3 hexagon plate. The $SAED$ pattern can be indexed as a 2-fold symmetry along the zone axis [0001], which demonstrates that the nanocrystal grow along [0001], with (0001) facet as top and bottom surfaces with a minimal distortion along $[01\bar{1}0]$ of the $(01\bar{1}0)$ facet and a significant distortion along $[10\bar{1}0]$ of the $(10\bar{1}0)$ facet. A selected area zoomed $HRTEM$ image (Fig. S4(d)) shows hexagonal lattice fringes with a lattice spacing of $d_{10\bar{1}0} \sim 3.462$ Å and

$d_{01\bar{1}0}$~4.022 Å, corresponding to the $(10\bar{1}0)$ and $(01\bar{1}0)$ plane, respectively. Further, the d-spacing observed in the fast Fourier transform of the HRTEM image (Fig. S4(e)) is consistent with the lattice spacing and the SAED pattern.

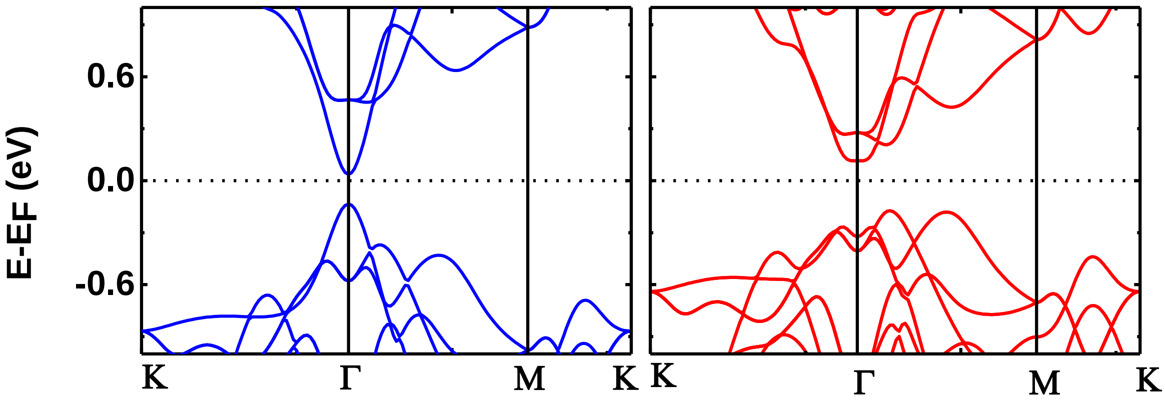

Fig. S4 Bulk band structure of Bi_2Se_3 (a) without and (b) with spin-orbit coupling.

Figure S4 shows the bulk band structure of hexagonal Bi_2Se_3 without spin-orbit coupling. The band structure shows parabolic valence band maximum (VBM) and conduction band minimum (CBM) at the Γ point, which confirms the direct band gap semiconducting behaviour of Bi_2Se_3. The band gap value is found to be $0.19\ eV$. On including the SOC, the band gap becomes indirect with an enhanced magnitude of $0.38\ eV$. The CBM and VBM at the Γ point become 'W' and 'M' shape due to the band inversion between the Bi and Se p_z orbitals.

References :

1. H. Zhang, C.-X. Liu, X.-L. Qi, X. Dai, Z. Fang, and S.-C. Zhang, Nat. Phys. 2009, **5**, 438-442.
2. L. Fu, and C. L. Kane, Phys. Rev. B 2007, **76**, 045302.
3. S. K. Das, and P. Padhan, ACS Appl. Nano Mater. 2020, **3**, 274–282.
